# Machine Learning Reduced Gene/Non-Coding RNA Features That Classify Schizophrenia Patients Accurately and Highlight Insightful Gene Clusters

**DOI:** 10.3390/ijms22073364

**Published:** 2021-03-25

**Authors:** Yichuan Liu, Hui-Qi Qu, Xiao Chang, Lifeng Tian, Jingchun Qu, Joseph Glessner, Patrick M. A. Sleiman, Hakon Hakonarson

**Affiliations:** 1 Center for Applied Genomics, Children’s Hospital of Philadelphia, Philadelphia, PA 19104, USA; liuy5@email.chop.edu (Y.L.); quh@email.chop.edu (H.-Q.Q.); changx@email.chop.edu (X.C.); tianl@email.chop.edu (L.T.); jingchun.qu789@gmail.com (J.Q.); glessner@email.chop.edu (J.G.); sleimanp@email.chop.edu (P.M.A.S.); 2 Division of Human Genetics, Department of Pediatrics, The Perelman School of Medicine, University of Pennsylvania, Philadelphia, PA 19104, USA; 3 Department of Human Genetics, Children’s Hospital of Philadelphia, Philadelphia, PA 19104, USA

**Keywords:** schizophrenia, machine learning, transcriptome, long non-coding RNAs

## Abstract

RNA-seq has been a powerful method to detect the differentially expressed genes/long non-coding RNAs (lncRNAs) in schizophrenia (SCZ) patients; however, due to overfitting problems differentially expressed targets (DETs) cannot be used properly as biomarkers. This study used machine learning to reduce gene/non-coding RNA features. Dorsolateral prefrontal cortex (dlpfc) RNA-seq data from 254 individuals was obtained from the CommonMind consortium. The average predictive accuracy for SCZ patients was 67% based on coding genes, and 96% based on long non-coding RNAs (lncRNAs). Machine learning is a powerful algorithm to reduce functional biomarkers in SCZ patients. The lncRNAs capture the characteristics of SCZ tissue more accurately than mRNA as the former regulate every level of gene expression, not limited to mRNA levels.

## 1. Introduction

Schizophrenia (SCZ) is a complex biological disorder that involves combined effect of many genes, each conferring a small increase in susceptibility to the illness [1]. The redundancy of the gene networks underlying SCZ indicates that many gene combinations have the potential to result in a brain dysfunction that can manifest as SCZ or a related neurodevelopmental disorder [2]. Next-generation sequencing (NGS) enables measures of the transcriptome gene expression through RNA-seq, however, expressed genes cannot be used as biomarkers in many diseases that involve complex genetic networks, due to high noise level from a large number of genes and small number of samples. While the current solution is considering only differentially expressed targets (DETs) between SCZ and healthy controls, this often has multiple potential problems. For example, single or a small number of differentially expressed genes may not be clinically important for SCZ [3,4], indicating that a more comprehensive analysis is necessary to reveal the underlying genetic network for SCZ. Selection criteria for DETs are arbitrary, and while many researchers use adjusted *p* value of 0.05 as a cut-off, this static standard often brings more ambiguity and neglects downstream analysis [5]. Even if only DETs were selected with a *p* value cut-off as features for labeling or prediction, the DET number could still be too large which means that an overfitting problem could exist theoretically [6]. The key to address overfitting from too many features is to effectively reduce the number of features.

Beside coding genes, non-coding RNAs, especially long non-coding RNAs (lncRNAs) are important factors in shaping SCZ networks and are dynamically regulated by neuronal activation [7,8,9,10], and should therefore also be considered as potential feature vectors for SCZ gene network regulations. In this study, we acquired dorsolateral prefrontal cortex (dlpfc) samples’ RNA-seq data from 254 subjects from the CommonMind consortium [11] (120 SCZ patients and 130 healthy controls, all non-Hispanic Caucasian). We then applied machine learning algorithms, including random forest, forward feature selection (ffs), and factor analysis to reduce the number of expressed genes into small list of feature vectors, in order to solve the overfitting problem. Two-fold shuffle tests showed that these selected feature vectors could accurately label SCZ patients versus controls. Selected genes were further clustered into gene modules through factor analysis, to explore potential functional units within the complex underlying genetic networks in SCZ.

## 2. Results

The statistical analysis and fold changes of genes were calculated. Altogether, 10,100 genes showed nominal significance with *p* < 0.05 uncorrected for multiple testing (Table S1. Among the 10,100, expression of 3483 genes were down-regulated, and expression of 6617 genes were up-regulated. Using the WebGestalt (WEB-based Gene SeT AnaLysis Toolkit) web tool [12], over-representation analysis (ORA) by the Reactome approach [13] highlighted genes involved in mitochondrial function as down-regulated; and genes involved in gene transcription as upregulated (Table S3 and Table S4).

### 2.1. Accuracy Measure for Labeling Schizophrenia (SCZ) Patients Based on 2-Fold Shuffle Testing

**Figure 1:**
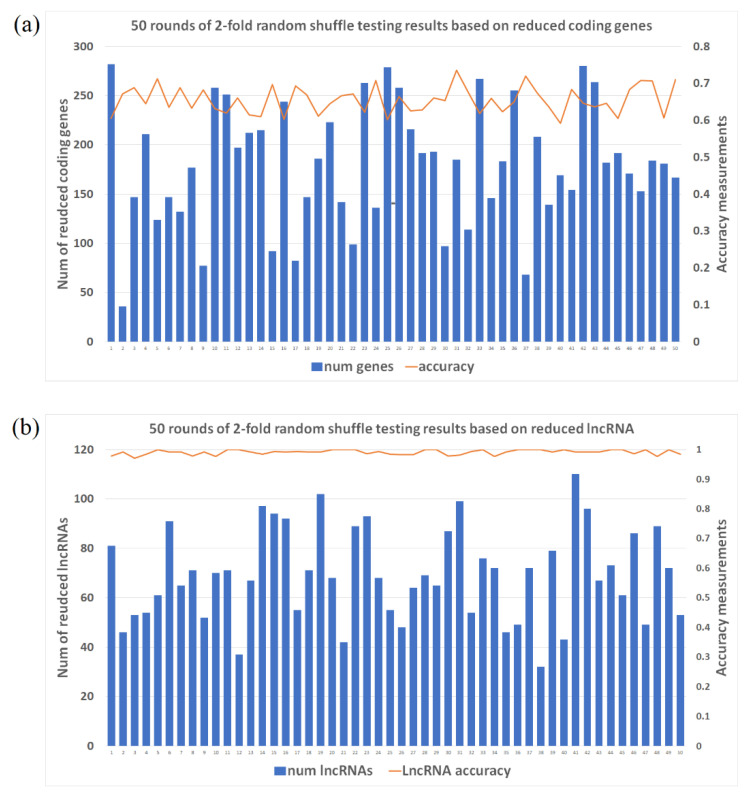
Two-fold random shuffle testing results for 50 rounds. *X*-axis is the round number, Y_1 axis (left) is the number of reduced genes (**a**) and lncRNAs (**b**), Y_2 axis (right) is the accuracy measurement ranged from 0 to 1.

As described in the method section, 2-fold shuffle testing was applied to test the labeling prediction 50 times. Reduced genes, based on multiple machine learning methods, ranged from 36 to 282, showed certain level of accuracy (~67%) in classifying SCZ patient’s dorsolateral prefrontal cortex (dlpfc) samples versus healthy controls (Figure 1a). In contrast, to acquire a similar accuracy, we needed at least 100 top expressed coding genes to do random forest classification, whereas the accuracy of the traditional K-means clustering based on the top differentially expressed coding genes was only 50.2% at best. K-means clustering is an unsupervised classification method without requiring an independent training dataset. It is the simplest and the most popular method when the number of clusters was known. But still overfitting is possible as we identified the differentially expressed genes in the same dataset as features used for K-means, which means that the accuracy of 50.2% by K-means is possibly over-estimated. The actual performance of K-means could be even worse than that of random forest. Machine learning methods reduced long non-coding RNAs (lncRNAs), ranging from 32 to 110 lncRNAs, and showing extremely high accuracy in classifying SCZ patients (accuracy level ~99%) (Figure 1b). In contrast, the accuracy of random forest classification based on the top differentially expressed lncRNAs was only ~64%, whereas the accuracy of the traditional K-means clustering based on the top differentially expressed lncRNAs was poor with the highest accuracy of 36.7%. These machine learning results, demonstrating high accuracy indicate the existence of an functionally essential regulation network for SCZ brain tissues. The key difference between the clustering methods is the feature vector selections, in other words, the selection of genes/lncRNAs representing the essential differences between two groups (SCZ vs. controls). K-mean clustering used the 100 top differentially expressed genes, which is the most routine method, instead of multiple filtering steps of our machine learning methods. Considering gene co-expression network, overlapped information from the 100 top differentially expressed genes is always a concern. More importantly, the nature and complexity of SCZ have determined that the disease is not impacted by single or several genes, and the most differentially expressed genes are not necessarily the essential regulators. For lncRNAs, the issue is even enlarged because lncRNAs work as regulators of networks instead of expressed genes.

### 2.2. Selected Gene/lncRNA Feature Based on Machine Learning Algorithm

**Figure 2:**
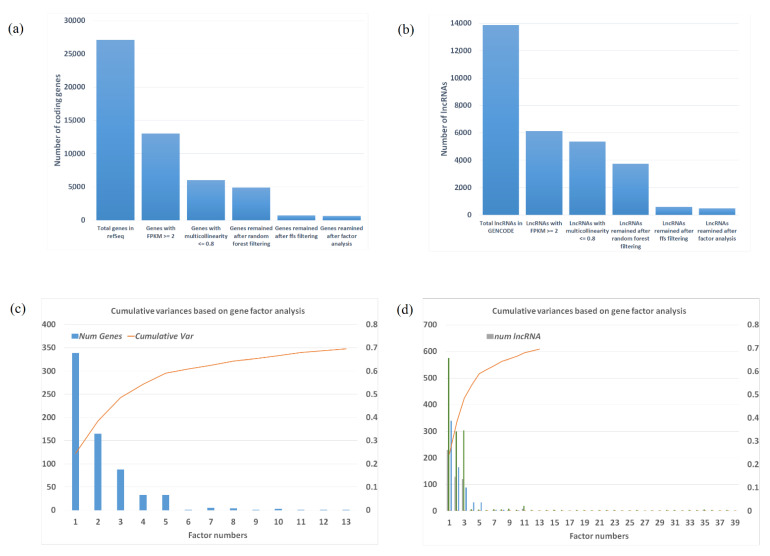
(**a**) Number of feature vectors for coding genes after multiple filtering methods; (**b**) number of feature vectors for lncRNAs after multiple filtering methods; (**c**) factor analysis cumulative curve and number of remain coding-gene feature vectors; (**d**) factor analysis cumulative curve and number of remain lncRNAs and their targeted genes’ feature vectors.

After multiple layers of filtering, including the machine learning methods, the number of genes reduced from 27,101 to 734 in the total of 254 dorsolateral prefrontal cortex (dlpfc) samples (Figure 2a). Expressions of all these genes have nominally statistical significance with *p* value range from 1.5 × 10^−9^ to 6.45 × 10^−3^. Among the 734 genes (Table S5), 412 were downregulated; and 322 genes were upregulated. These genes were found to be enriched in glycosaminoglycan metabolic processes (adjusted *p* value = 3.1 × 10^−3^), aminoglycan metabolic processes (adjusted *p* value = 3.7 × 10^−3^), mucopolysaccharide metabolic processes (adjusted *p* value = 1.2 × 10^−2^), based on Gene Ontology [14]. A total of 13,871 long non-coding RNAs (lncRNAs) identified in GENCODE were reduced to 605 using comparable pipelines. The results suggest that by combining multiple machine learning methods is a powerful tool to reduce the number of gene features which represent the variations of the data. It is also worth mentioning that only 255 of the 734 coding genes have differential expression with False Discovery Rate (FDR) Adjusted *p* value < 0.05.

### 2.3. Factors and Potential Gene Modules from Factor Analysis

**Figure 3:**
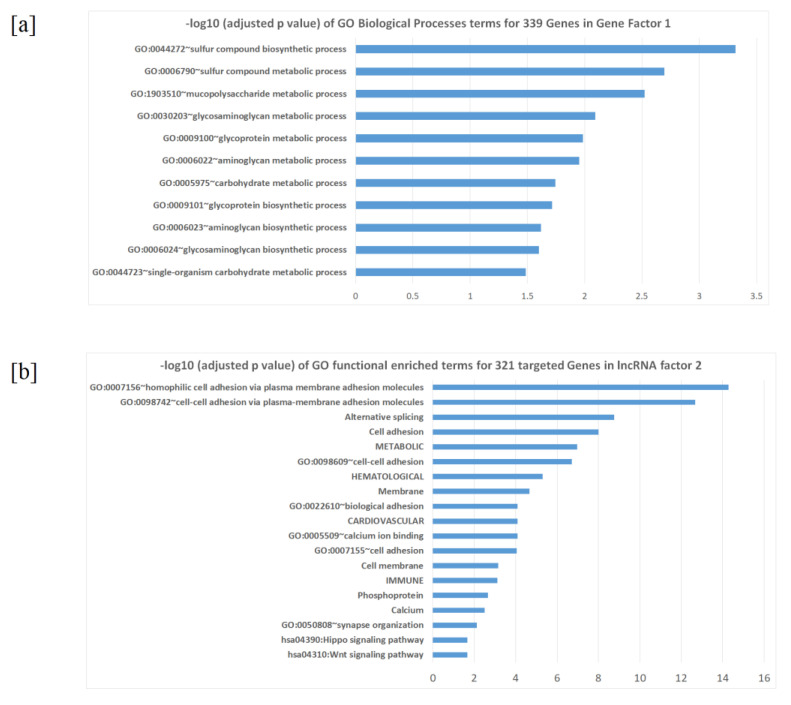
-Log10 (Adjusted *p* value) scale for enriched functional pathways: (**a**) gene factor 1; (**b**) targeted genes in lncRNA factor 2.

The purpose of factor analysis is to group the reduced feature vectors genes/lncRNAs into modules, which could function together for a complex disease as SCZ. The 734 genes were clustered into 66 factors, where the first 13 factors contribute ~70% of the variances (Figure 2c, Table S5). Factor 1 contains 339 genes and contributes 24.5% of the total variance, all genes in factor 1 are down regulated in SCZ versus health controls, as shown by differential expression in CommonMind consortium database [15]. Not unexpectedly, the over-representation analysis (ORA) by Human Phenotype Ontology (HPO) shows that factor 1, as the major factor of SCZ transcriptome, contains a number of genes critical in neurodevelopment that are involved in various brain disorders, including HP:0001298_Encephalopathy, HP:0001098_Abnormal fundus morphology, and HP:0004329_Abnormal morphology of the posterior segment of the globe. Enrichment analysis by biological pathways using the expressed gene list (FPKM ≥2) as the background (Figure 3a) highlighted sulfur compound biosynthetic processes (FDR adjusted *p* value = 4.8 × 10^−4^), glycosaminoglycan metabolic processes (FDR adjusted *p* value = 8.1 × 10^−3^), and glycoprotein metabolic processes (FDR adjusted *p* value = 0.019). Eight genes were identified in previous PG2 GWAS studies [16], including *APH1A* (rs140505938, FDR adjusted *p* value = 4.49 × 10^−10^), *ASPHD1* (rs11646127, FDR adjusted *p* value = 4.55 × 10^−11^), *BRINP2* (rs6670165, FDR adjusted *p* value = 4.45 × 10^−8^), *CHRM4* (rs7951870, FDR adjusted *p* value = 1.26 × 10^−11^), *INO80E* (rs11646127, FDR adjusted *p* value = 4.55 × 10^−11^), *PCCB* (rs7432375, FDR adjusted *p* value = 7.26 × 10^−11^), *SPCS1* (rs3617, FDR adjusted *p* value = 4.26 × 10^−11^), and *TAC3* (rs61937595, FDR adjusted *p* value = 2.02 × 10^−12^), all these hotspots were also identified in CLOZUK, which SCZ cases were ascertained with the assistance of Novartis, and the samples consisted of individuals with treatment-resistant schizophrenia according to the clozapine registration forms completed by treating psychiatrists [17]. Of note, 159 out of the 339 genes (46.9%) have at least one supportive evidence from previous knowledge, and 12 genes have at least four supportive evidences (Table 1).

A total of 166 genes clustered into factor 2 and majority of them (94%) are up regulated in SCZ. Factor 2 contributes 13.9% of the data variance (Figure 2c, Table S6), 11 genes (6.7%) were found to be differentially expressed and none of them are down regulated in SCZ. A total of 65 out of 165 genes (39.3%) have at least one supportive evidence from previous knowledge, and 5 genes have at least four supportive evidences (Table 1). Enrichment analysis by biological pathways highlighted phosphorylation (FDR adjusted *p* value = 5.1 × 10^−3^). There are 88, 33, and 33 genes in factor 3, 4 and 5 (Table S6), respectively, contributing 10%, 5.7% and 4.8% of the variance, respectively.

For lncRNAs, factor analysis resulted in 91 factors, where the first 45 factors (contain 496 lncRNAs) explain ~70% of the variations of the expression data (Figure 2d, Table S7). The first 3 factors of lncRNA factor analysis explained 21%,14%, and 13% variance respectively, with a subtotal of 48%. Among the 496 lncRNAs, only 88 have differential expression with FDR Adjusted *p* value < 0.05. The genes closest to the lncRNA binding sites were identified as their target genes. Mapping lncRNAs and their targets is a challenging problem. Computational methods cannot effectively solve the false positive issue, and literature-based databases usually have low sensitivity. Previous studies [18,19,20] showed that lncRNAs tend to regulate expression of neighboring protein-coding genes and thus contribute to the regulation of mRNA and protein levels in mammal. Therefore, choosing the closest genes of the lncRNA could capture at least a certain level of corresponding target genes and be more practical in the application. However, due to gene functional overlapping, choosing only the closest genes may indeed introduce bias. In the trade-off between high sensitivity and acceptable specificity, based on our results and current literature, three closest genes is plausibly the most reasonable choice of the selection.

A total of 1193 genes were selected and found to be enriched in multiple disease category [21], METABOLIC (FDR adjusted *p* value = 2.8 × 10^−5^), CARDIOVASCULAR (FDR adjusted *p* value = 3.2 × 10^−2^), HEMATOLOGICAL (FDR adjusted *p* value = 7.3 × 10^−3^), IMMUNE (FDR adjusted *p* value = 4.8 × 10^−2^), and post translational modifications such as phosphorylation (8.2 × 10^−4^). Multiple neurodevelopmental related pathways which cannot be captured by the mRNA pathway analysis, are highlighted for the lncRNAs’ targeted gene sets, including regulation of transferase activity (FDR adjusted *p* value = 1.8 × 10^−3^), neuron projection morphogenesis (FDR adjusted *p* value = 4.8 × 10^−3^), positive regulation of nervous system development (FDR adjusted *p* value = 4.6 × 10^−3^); regulation of kinase activity (FDR adjusted *p* value = 5.7 × 10^−3^), neuron differentiation (FDR adjusted *p* value = 8.3 × 10^−3^), and neuron projection development (FDR adjusted *p* value = 8.8 × 10^−3^). Target genes in lncRNA factors with high supportive evidence are shown in Table 2.

Consequently, we tested the machine learning algorithm in an independent dataset including 22 non-EA SCZ and 27 controls. The performance with coding genes was highly reproducible with the accuracy of 95%, whereas the accuracy with lncRNAs was only 37%.

## 3. Discussion

Growing evidence indicates that distinct neuronal ncRNAs, particularly lncRNAs, are likely to influence the development of neurodevelopmental diseases, including SCZ [7]. However, to date, neither genes in neurodevelopmental networks nor the lncRNAs in regulation processes have been successfully applied as feature vectors to label the phenotypic status of the patients. The main obstacle is the number of genes/lncRNAs as predictive features is huge (over 20,000 genes and 10,000 lncRNAs), and the number of biological samples, especially brain tissues, is usually small due to the difficulty in sample collections. Therefore, predictive models are deemed to fail due to overfitting issues. Currently, the solution for this problem is to select a small number of genes that are differentially expressed between SCZ and controls. While this may help, the algorithm and criteria used for selecting these gene remains controversial, and genes contributing to the network with small effects and less statistical significance may be missed.

Machine learning methods have been proven to be effective in reducing the feature vectors while capturing essential data differences in studies of many fields, include genetic expression studies [22,23]. In this study, we applied multiple machine learning layers for expressed genes in dorsolateral prefrontal cortex (dlpfc) RNA-seq data from 254 samples from the CommonMind consortium, in order to show that reduced gene/lncRNA features could accurately classify SCZ patients versus healthy controls. Combining machine learning methods, such as random forest and forward feature selection (ffs) for expressed genes/lncRNAs, the number of genes was significantly reduced from over 25,000 to an average ~180 genes while the lncRNA number was reduced from 13,000 to on average ~70 through the simulations. The 2-fold shuffle tests (samples split to 1:1 ratio, half used as training data and rests used as testing data) applied in 50 separate rounds (Figure 1) shows that reduced gene feature vector has modest power (~67% accuracy) in classifying SCZ patients versus healthy controls, whereas lncRNAs could serve as an effective predictor (~99% accuracy). On the other hand, the random forest classification tends to require a larger number of top differentially expressed coding genes to get similar accuracy as that of machine learning selected genes, as top differentially expressed coding genes may not be independent of each other because of gene co-expression networks. The random forest classification based on top differentially expressed lncRNAs has poor performance, while with lncRNAs as regulators highly differential lncRNAs may not be the most important lncRNAs. These results demonstrate that machine learning has the potential to be an alternative methods to detect the essential differences of gene expressions in SCZ, also the regulation networks involved in lncRNAs are more stable than gene expression networks, in other words, gene expressions remain highly diverse for different persons but the lncRNAs expression seems universal among different individuals. More importantly, as demonstrated by our findings that the average predictive accuracy for SCZ patients is 67% based on coding genes, and 96% based on lncRNAs, lncRNAs represent a more accurate biomarker for the SCZ transcriptome. The lncRNAs regulates every level of gene expression, including but not limited to mRNA levels, which may explain why lncRNAs capture the characteristics of SCZ tissue more accurately [24]. Considering gene co-expression network, duplicated/overlapped information from top differentially expressed mRNAs is always a concern. On the other hand, too many mRNAs will cause overfitting unavoidably. Our study, therefore, suggests lncRNAs are more informative and better features.

**Figure 4:**
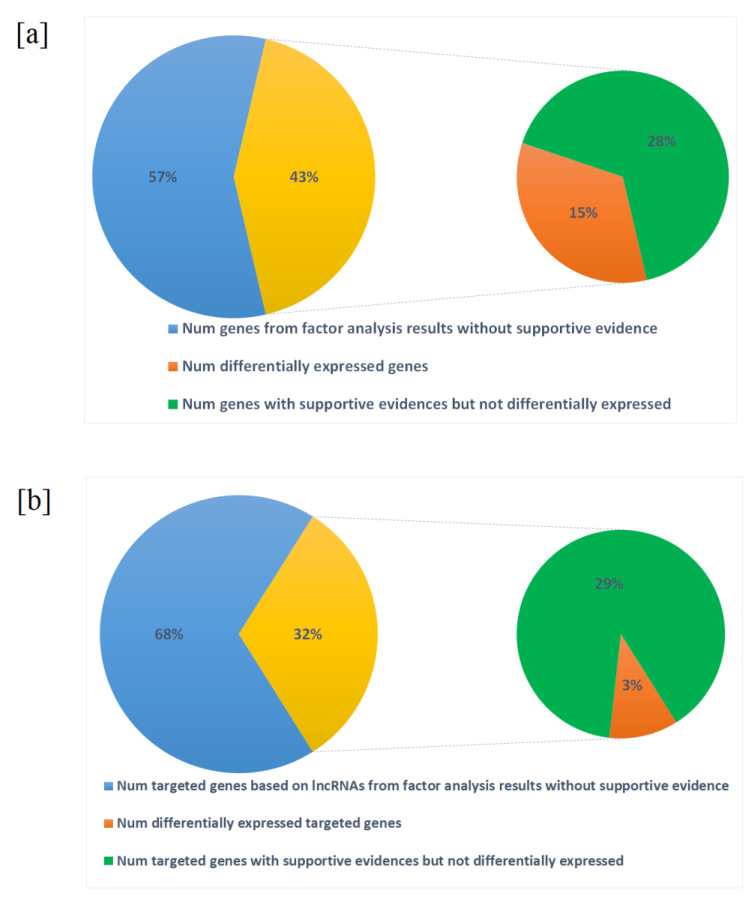
Portion of differentially expressed genes/lncRNAs versus genes/lncRNAs with at least one supportive evidence: (**a**) coding genes after factor analysis; (**b**) targeted coding genes based on lncRNA genomic locus.

Furthermore, the reduced gene/lncRNA features were clustered based on factor analysis to form the gene modules. Our study showed that major factors were enriched for genes important in neurodevelopment and brain disorders, thereby proving the validity of the dimension reduction process. While genes in major factors show enrichment of SCZ related pathways or neurodevelopmental associated network and can serve as a proof-of-principle of this study, other factors may harbor novel knowledge about SCZ and warrant further study. Genes within each factor have higher portion of SCZ supportive evidences while known differentially expressed genes only counted small portion of genes in each factor (Figure 4). Combining these clues together indicates that the machine learning models capture contributing genes more effectively compared to traditional differential expression tests. SCZ genetics involves combined effect of many genes, each conferring a small increase in susceptibility to the illness [25].

Genes in certain factors highlighted SCZ-associated networks and the biochemical molecules, synthesis/metabolism, as neuro system modulators. For example, 339 genes in gene factor 1 were enriched in sulfur compound biosynthetic and metabolic processes (adjusted *p* value = 4.8 × 10^−3^). Sulfur is an essential chemical for proteinogenic amino acid methionine (Met), and methionine-folate cycle-dependent one-carbon metabolism is implicated in the pathophysiology of SCZ while deficiencies in the one-carbon metabolism components folate is consistent findings in SCZ patients [26]. These genes are also enriched in glycosaminoglycan metabolic processes (adjusted *p* value = 8.1 × 10^−3^). Glycosaminoglycans, an alternative name of mucopolysaccharides, play critical roles in the normal function of the central nervous system [27]. Abnormal glycosaminoglycan synthesis in cerebral cortex have been reported to associated with SCZ [28]. Hyaluronic acid, one of the major classes of glycosaminoglycans, is a critical component on the surface of perineuronal nets (PNNs), while a decreased number of PNNs is associated with schizophrenia [29]. Other enriched chemical synthesis procedures for genes in factor 1 include glycoprotein metabolic processes (adjusted *p* value = 0.019) where *p*-glycoprotein a major efflux pump in the blood-brain barrier, has a profound effect on entry of drugs, peptides and other substances into the central nervous system [30]. For lncRNA factor 2, 321 targeted genes (the union of 128 lncRNAs’ top three closest genes) were significantly enriched in cell adhesion (adjusted *p* value = 9.7 × 10^−9^), phosphorylation (adjusted *p* value = 2.2 × 10^−3^), SCZ associated pathways include calcium ion binding, while dysregulation of neural calcium has been found signaling in SCZ [31] (adjusted *p* value = 8.1 × 10^−5^) as well as the Wnt signaling pathway, a crucial pathway in neurodevelopment and in regulating the function and structure of the adult nervous system [32] (adjusted *p* value = 0.022) (Figure 3b). In contrast, many factors although with high consistent regulation tendency have no known genetic functions. For example, gene factor 2 (94% genes up regulated in SCZ, 39.3% have at least one supportive evidence, and only 11% of the genes are known to be differentially expressed in SCZ), gene factor 3 (78% genes up regulated in SCZ, 31.8% have at least one supportive evidence, and only 4.5% of the genes are known to be differentially expressed), gene factor 4 (70% genes down regulated in SCZ, 42.4% have at least one supportive evidence, and only 15% of the genes are known to be differentially expressed), and lncRNA factor 1, which contains 229 lncRNAs (597 target genes) but no enrichment were identified for biological functions. Taken together, these factors are potential targets for researchers to explore in further studies. One of the limitations of this study is the selection of target genes. We chose 3 closest genes of a lncRNA to capture the corresponding target genes based on previous studies showing that lncRNAs tend to regulate the expression of neighboring protein-coding genes [18,19,20]. The mapping procedure of lncRNAs to their target genes is currently a challenging problem and without a gold standard. We chose the 3 closest genes of a lncRNA to balance the trade-off between high sensitivity and acceptable specificity. More sophisticated approaches are still warranted. In addition, we emphasize that, as an exploratory study with limitations, the prediction model created in this study warrants further validation in other datasets, especially in different ethnicities. As shown by our data in a small sample of non-EA individuals from the CommonMind database (including 22 SCZ and 27 controls), the prediction accuracy of coding genes was reproducible, but not that of lncRNAs. In contrast to coding genes directly determining the pathophysiology of SCZ, lncRNAs have only regulatory and indirect effects in SCZ. Their prediction performance might thus be inferior if applied to a different ethnicity.

## 4. Methods & Materials

### 4.1. RNA-Seq Data for Dorsolateral Prefrontal Cortex (DLPFC) Samples

This study was approved by The Children’s Hospital of Philadelphia (CHOP) Institutional Review Board (IRB). All the patients who participated in this project were consented and they agree to the publication of the results. RNA-seq data of dorsolateral prefrontal cortex (DLPFC) samples were obtained from the CommonMind consortium [11]. More specifically, the RNA-seq data were downloaded from the CommonMind Consortium Knowledge Portal at synapse (https://www.synapse.org/#!Synapse:syn2759792/wiki/69613) accessed on 1 January 2017.

To minimize the confounding effect of ethnicity, we only selected SCZ patients and controls who are of European ancestry (EA). A total of 254 RNA-seq BAM files were obtained include 120 SCZ patients and 134 healthy controls. Samples with a minimum of 50 million mapped reads and less than 5% rRNA-aligned reads were retained for downstream analysis. The RNA-seq data were aligned using the Spliced Transcripts Alignment to a Reference (STAR) [33]. Details for individuals, such as gender, age, and read counts/unique reads were listed in Table S2.

Based on the consortium’s description, RNA was isolated from 50 mg homogenized tissue in Trizol using the RNeasy kit based on the instructional protocol. The mean total RNA yield was 15.3 µg (±5.7). The RNA integrity number (RIN) was determined by fractionating RNA samples on the 6000 Nano chip (Agilent Technologies, Santa Clara, CA, USA) on the Agilent 2100 Bioanalyzer. The mean RIN was 7.7 (±0.9), and the mean ratio of 260/280 was 2.0 (±0.02). Processing order was re-randomized prior to ribosomal RNA (rRNA) depletion. Briefly, rRNA was depleted from about 1 µg of total RNA using a Ribo-Zero Magnetic Gold kit (Illumina/Epicenter Cat # MRZG12324) to enrich for polyadenylated coding RNA and non-coding RNA. The sequencing library was prepared using the TruSeq RNA Sample Preparation Kit v2 (RS-122–2001-48 reactions) in batches of 24 samples. A pool of 10 barcoded libraries was layered on a random selection of two of the eight lanes of the Illumina flow cell bridge amplified to ~250 million raw clusters. One-hundred base pair paired end reads were obtained on a HiSeq 2500. The sequence data were processed for primary analysis to generate Quality Control (QC)values.

### 4.2. Gene/Non-Coding RNAs Expression Matrix

The genomic template used for coding genes expressions is hg19 refSeq, and long non-coding RNAs template is GENCODE version 19 [34]. The expression matrix was generated based on Cuffnorm functions in Cufflink package version 2.2.1 [35], and the SCZ and controls groups are normalized. More specifically, the Cuffnorm reports expression values in Fragments Per Kilobase (FPKM) for each gene were properly normalized based on library size. To eliminate potential noisy signals, the gene expression FPKM values less than 2 were removed due to potential noises from low FPKM genes. Genes/lncRNAs with collinearity over 80% were removed because generally feature selection methods assume feature vectors are independent to each other. Differential expression analysis was undertaken by independent Student’s *t*-tests based on the FPKMs after the classic-FPKM normalization.

### 4.3. Gene Reductions Using Machine Learning Algorithms

Multiple machine learning algorithms include random forest, forward feature selection (ffs), and factor analysis, were applied to select and reducing the informative gene/lncRNA features between SCZ and controls. Random forest is one of the most widely used algorithms for feature selection, which computes relative importance or contribution of each gene feature in the prediction, then scales the relevance down so that the sum of all scores is 1. All the genes/lncRNAs with zero relative importance were removed. The following parameters were applied for the random forest model, including: the function to measure the quality of a split, using “gini”; the minimum number of samples required to split an internal node equals 2; and nodes are expanded until all leaves are pure or until all leaves contain less than 2. The number of features to consider when looking for the best split equals the square root (num_features) and the number of trees in the forest equals 500.

The second algorithm forward feature selection (ffs) is one of the most common methods to reduce number of features for machine learning inputs by trying to find the best features which improve the performance of the model. The modeling codes are based on based on the Scikit-learn package (version 0.21.3) in Python language [36].

In order to test the predictive abilities for selected gene/lncRNA features, we applied a 2-fold shuffle testing. In other words, the SCZ and control samples were split into 1:1 ratio for 50 rounds randomly, one set used as training data and another one used as independent testing set. Gene/lncRNA features were selected as described in previous paragraph for training data (to overcome overfitting problem, the only parameter altered is we required random forest relative importance >0.0005 rather than >0), then a random forest classifier is applied to label whether the sample is SCZ or control in testing data based on training data gene/lncRNA features.

Factor analysis was applied to the entire sample set for further clustering gene/lncRNA features. Factor analysis is a statistical method used to describe variability among observed, correlated variables in terms of a potentially lower number of unobserved variables called factors, and the methods have been proven to be a good interpreter for gene networks and pathways [37,38]. The number of factors chosen in the model was 50 for both coding genes and lncRNAs based on Kaiser–Meyer–Olkin (KMO) test (when eigenvalues are greater than one), and the rotation method, “varimax”. The Python-based factor_analyzer package was used in the analysis (version 0.3.1).

For validation, we further tested the machine learning algorithm in an independent dataset, i.e., a small sample of non-EA individuals available from the CommonMind database (including 22 SCZ and 27 controls).

Supportive evidences for SCZ genes were collected based on SZDB, a database contains various layers of data of schizophrenia researches, such as genetic data, copy number variants (CNVs) data, whole genome/exome sequencing (WGS/WES) data, gene expression data, functional genomics data, and protein–protein interaction data [39].

## Figures and Tables

**Table 1:** Genes in gene factors with high supportive evidences, including genome-wide association study (GWAS), Genome Wide Linkage Study (Linkage) Copy Number Variation (CNV), integrative analysis (Integrative), differentially methylated (Diff Methy), Differentially expressed (Diff Exp), identified by exome sequencing (Exome), expression level in brain tissues (Brain Exp), Gene Ontology (GO), and total score (Score)

Factor	Gene	GWAS	Linkage	CNV	Integrative	Diff Methy	Diff Exp	Exome	Brain Exp	GO	Score
1	ASPHD1	1	0	1	0	0	1	1	1 (26.68)	0	5
1	AK4	0	0	0	0	1	1	0	1 (13.55)	1	4
1	APH1A	1	0	0	0	0	0	1	1 (43.24)	1	4
1	FPGS	0	0	0	0	1	1	0	1 (14.38)	1	4
1	FSCN1	0	0	0	0	1	1	1	1 (48.13)	0	4
1	INO80E	0	0	1	1	1	0	0	1 (20.22)	0	4
1	P2RX6	0	0	1	0	1	1	0	0 (4.99)	1	4
1	PCCB	1	0	0	1	1	0	0	1 (26.64)	0	4
1	PRODH	0	1	0	0	0	1	1	1 (24.04)	0	4
1	SCN1B	0	0	0	0	1	1	0	1 (25.12)	1	4
1	SEMA7A	0	0	0	0	1	1	0	1 (13.64)	1	4
2	BCCIP	0	0	0	0	1	1	1	1 (26.92)	1	5
2	HNRNPU	0	0	0	0	1	1	1	1 (108.84)	0	4
2	HSP90AA1	0	0	0	0	0	1	1	1 (411.41)	1	4
2	NRG1	0	1	0	0	1	0	1	0 (4.47)	1	4
2	PDE4B	1	1	0	0	0	0	0	1 (38.88)	1	4
3	TIMP2	0	0	0	0	0	1	1	1 (107.98)	1	4
7	BCL6	0	0	0	0	1	1	1	1 (29.50)	1	5
9	RERE	1	0	0	1	0	0	0	1 (25.51)	1	4

**Table 2:** Target genes in lncRNA factors with high supportive evidence.

Factor	lncRNA	Target Gene	GWAS	Linkage	CNV	Integrative	Diff Methy	Diff Exp	Exome	Brain Exp	GO	Score
1	ENSG00000247735.2	ASPHD1	1	0	1	0	0	1	1	1 (26.68)	0	5
1	ENSG00000232912.1	RERE	1	0	0	1	0	0	0	1 (25.51)	1	4
1	ENSG00000235770.1	FN1	0	1	0	0	0	0	1	1 (38.33)	1	4
1	ENSG00000235831.2	ITPR1	0	0	0	0	1	0	1	1 (25.59)	1	4
1	ENSG00000239569.2	SRPK2	1	0	0	0	1	0	0	1 (75.17)	1	4
1	ENSG00000243762.1	RANBP1	0	1	1	0	1	0	0	1 (30.73)	0	4
1	ENSG00000247735.2	SEZ6L2	1	0	1	0	0	0	0	1 (38.77)	1	4
1	ENSG00000257126.1	FOXG1	1	0	0	0	1	0	0	1 (22.05)	1	4
1	ENSG00000261220.2	ST3GAL1	0	0	0	0	1	0	1	1 (8.93)	1	4
1	ENSG00000271849.1	PJA2	0	0	0	0	1	0	1	1 (144.54)	1	4
2	ENSG00000224563.1	BCL6	0	0	0	0	1	1	1	1 (29.50)	1	5
2	ENSG00000226978.1	MAGI2	0	1	0	0	1	0	0	1 (24.46)	1	4
2	ENSG00000236031.1	AKT3	1	0	0	0	0	0	1	1 (35.93)	1	4
2	ENSG00000248816.1	TENM3	0	0	0	0	1	1	0	1 (8.06)	1	4
2	ENSG00000272367.1	RASA1	0	0	0	0	1	1	0	1 (20.45)	1	4
3	ENSG00000272989.1	DLG1	0	0	1	0	0	1	1	1 (52.38)	1	5
3	ENSG00000239569.2	SRPK2	1	0	0	0	1	0	0	1 (75.17)	1	4
3	ENSG00000273164.1	PRODH	0	1	0	0	0	1	1	1 (24.04)	0	4
3	ENSG00000273164.1	DGCR2	0	1	1	0	0	0	1	1 (30.80)	0	4
37	ENSG00000248816.1	TENM3	0	0	0	0	1	1	0	1 (8.06)	1	4

## Data Availability

The data presented in this study are available in the Supplementary Materials.

## Supplementary Materials

The following are available online at https://www.mdpi.com/1422-0067/22/7/3364/s1, Table S1. The statistical analysis and fold changes of gene expression; Table S2. Details of the research subjects; Table S3. Downregulated genes pathways; Table S4. Upregulated genes pathways; Table S5. Selected Gene features based on Machine learning algorithm; Table S6. Factors of coding genes; Table S7. lncRNAs.
